# Recognizing Sacral Insufficiency Fractures Hidden in Plain Sight: An Illustrative Case Report

**DOI:** 10.7759/cureus.108982

**Published:** 2026-05-16

**Authors:** Jose Valerio, Andrea V Maraone, Noe Santiago Rea, Patricia del Carmen Bergna Vazquez, Jorge Zumaeta

**Affiliations:** 1 Neurological Surgery, Palmetto General Hospital, Hialeah, USA; 2 Neurosurgery, Miami Neuroscience Center at Larkin, Miami, USA; 3 Neurosurgical Oncology, Latinoamerica Valerio Foundation, Hialeah, USA; 4 Neurology, Palmetto General Hospital, Hialeah, USA; 5 Neurosurgery, Hospital Juárez de México, Mexico DF, MEX; 6 Neurosurgery Service, Hospital Nacional Arzobispo Loayza, Lima, PER; 7 Neurosurgery, Hospital Nacional Guillermo Almenara Irigoyen, Lima, PER

**Keywords:** magnetic resonance imaging, osteoporosis, pelvic fracture, sacral insufficiency fracture, transsacral screw fixation

## Abstract

Sacral insufficiency fractures (SIFs) are stress fractures that occur when normal biomechanical forces act on weakened bone, most commonly in elderly patients with osteoporosis or other conditions that compromise bone integrity. Because symptoms are often nonspecific, typically presenting as low back, sacral, or pelvic pain, SIFs are frequently underdiagnosed or misinterpreted as other pathologies, including lumbar spine disorders or metastatic disease. Conventional radiographs have low sensitivity, and although computed tomography (CT) can help identify fracture lines and assist in surgical planning, magnetic resonance imaging (MRI) remains the most sensitive modality for early detection. We present two cases of SIFs with progressive pain and functional impairment. The first case involved an 86-year-old woman who developed severe low back pain following a fall, with imaging revealing bilateral sacral fractures involving Denis zones I and III. The second case involved a 61-year-old woman with a prior history of lumbar fusion who presented with worsening lumbar pain radiating to the lower extremities, saddle numbness, and fecal incontinence; imaging demonstrated bilateral sacral fractures with displacement. Both patients had persistent symptoms and fracture instability despite conservative measures and underwent minimally invasive bilateral sacroiliac fusion with trans-sacral screw fixation. Postoperatively, both patients experienced significant pain reduction and improved functional status. These cases highlight the importance of maintaining a high index of suspicion for SIF in patients with unexplained lumbosacral pain and risk factors for bone fragility. While conservative management remains the first-line treatment, surgical stabilization with trans-sacral screw fixation may provide effective mechanical stability, rapid pain relief, and improved functional recovery in patients with unstable fractures or failure of nonoperative therapy.

## Introduction

The sacrum acts as a crucial support structure for the spine, connecting it to the pelvic ring and distributing biomechanical forces. Minor misalignments in this area can lead to significant symptoms, making pelvic incidence key for predicting surgical outcomes in sacral fractures [1]. 

Sacral insufficiency fracture (SIF) is a type of stress fracture that occurs when normal stresses exceed the bone's decreased mineralization and elastic resistance [2-5]. Diagnosis is usually delayed or missed, since its symptoms are nonspecific (vague to severe back pain) and can mirror different pathological entities [4,6-9].

Diagnosing SIF can be challenging; the best approach after the physical examination is at least an anteroposterior (AP) X-ray of the pelvis [10]; sometimes the fracture is not visible, and computed tomography (CT) scans can be valuable for diagnosing sacral fractures and for surgical planning [11]. Magnetic resonance imaging (MRI) is the most accurate imaging technique to diagnose SIF and can detect nerve compression [11-13]. 

Sacral fractures in young people and older adults are becoming more common. However, with the relatively low incidence and complexity of the fracture patterns [14], many doctors have limited experience with them, which can lead to misdiagnosis or inadequate treatment. Therefore, understanding sacral fractures is crucial for ensuring proper diagnosis and management, which could range from conservative management to surgical options [4,6,9,13,15,16]. 

This manuscript aims to aid the readers in identifying and diagnosing SIF and, likewise, to demonstrate that trans-sacral screw fixation is the best surgical option since it decreases the morbidity and mortality in patients with SIF.

## Case presentation

Case 1

**Figure 1:**
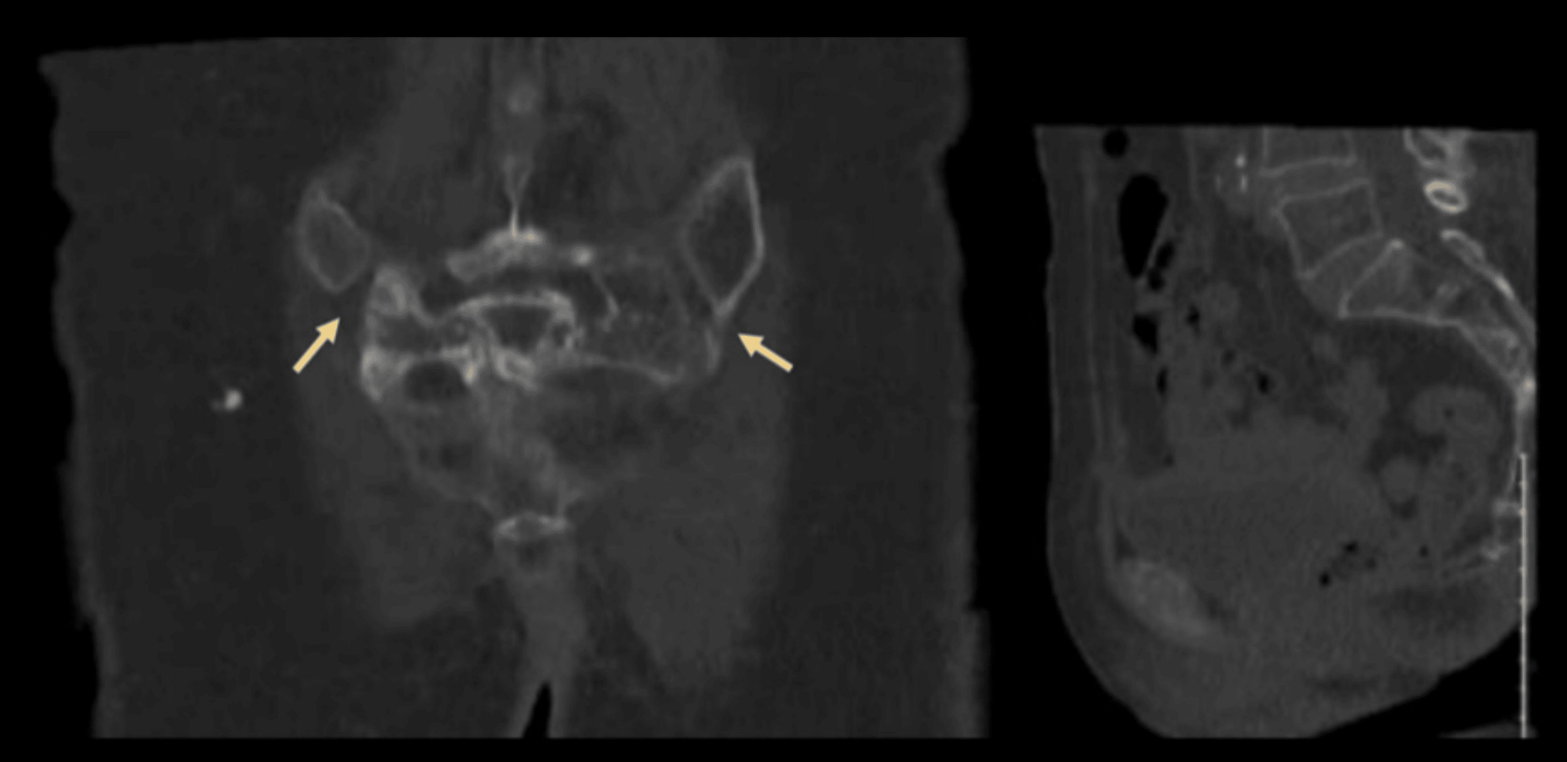
The pelvic CT scan reveals nondisplaced bilateral sacral fractures adjacent to the sacroiliac joints (yellow arrows), accompanied by a mild fracture of the anterior cortex of S2. Spondyloarthritic changes are also noted in the lumbar spine.

**Figure 2:**
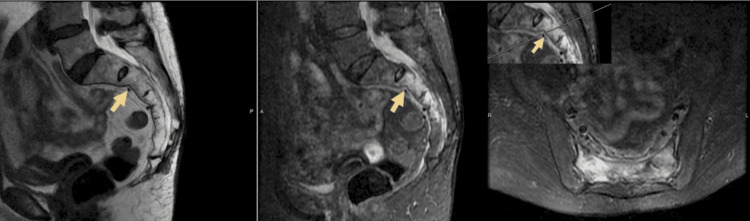
Moderate bilateral sacral edema is observed due to nondisplaced insufficiency fractures, with more pronounced swelling on the right side compared to the left. Sagittal images show a fracture in the anterior cortex of the S2 vertebra (yellow arrows). No hip fractures are present. Additionally, there is evidence of a previous open reduction and internal fixation surgery on the left femur.

An 86-year-old female with no significant medical history presented with lower back pain following a fall two weeks prior. The patient tripped and landed on her buttocks, leading to lower back pain 9/10, radiating to the right leg. On examination, she was alert and oriented. She had full motor strength in both upper and lower extremities, preserved sensation, and 1/4 reflexes in both upper and lower extremities. Tenderness was noted over the lower back and right hip, and sphincter control was intact. Pelvic X-ray examinations revealed no significant findings. A hip CT (Figure 1) showed nondisplaced bilateral sacral fractures and a minor fracture of the anterior cortex of S2. The pelvic MRI (Figure 2) provided a clearer view of fracture signs at the S2 level.

**Figure 3:**
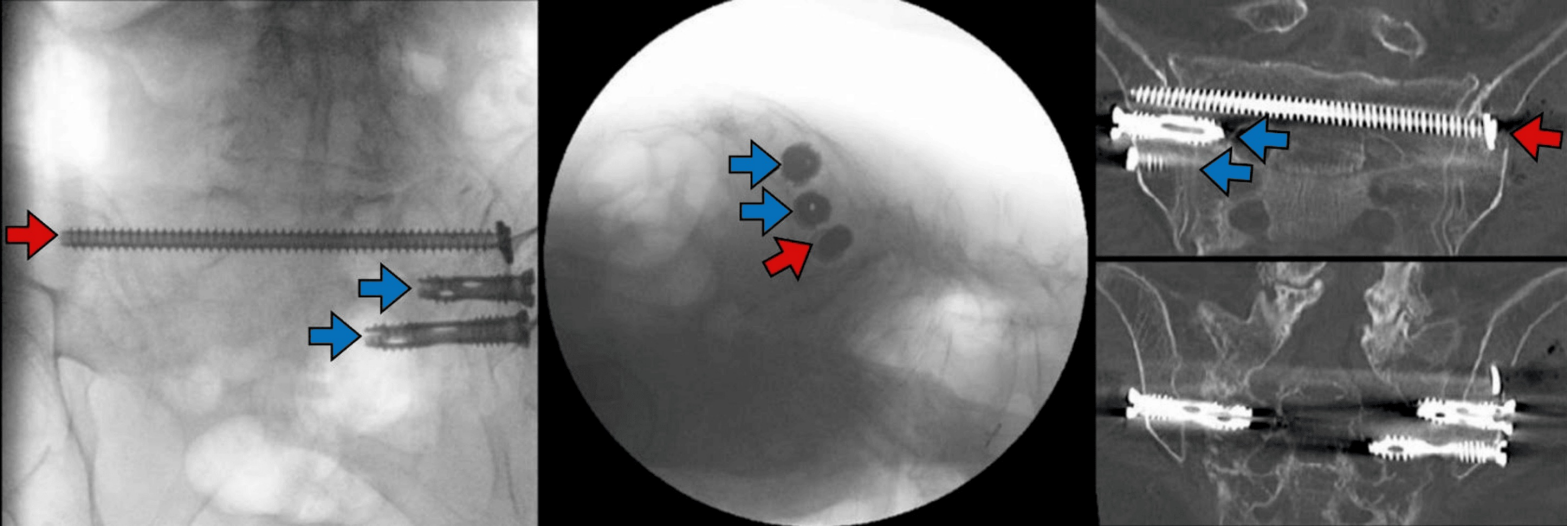
The first image illustrates the initial stage of the surgery, featuring the placement of a trans-sacral iliac screw (red arrow) along with two screws for the fusion of the left sacroiliac joint (blue arrows). The second image presents the final sagittal view displaying bilateral sacroiliac screws and the trans-sacral iliac screw positioned at the S1 level. The third image presents the final coronal view.

Due to unstable SIFs involving zones 1 and 3, surgical intervention was recommended. The patient underwent bilateral sacroiliac joint fusion and trans-sacral screw placement. Immediate control imaging studies (Figure 3) were done after the surgical procedure. Two days post-surgery, the patient reported a pain score of 6/10 and was able to move all four extremities against gravity. Sensation, reflexes, and sphincter control were all normal. The surgical site dressing was clean and dry, and while the patient experienced localized pain at the surgical site, there were no neurological deficits. She was discharged home with instructions to use a sacroiliac joint belt and engage in physical therapy. 

At her first follow-up, 15 days after surgery, the patient was asymptomatic, with well-healed surgical sites and no signs of infection or wound dehiscence. She reported a favorable response to the procedure, and her next follow-up was scheduled for one month after surgery.

Case 2

A 61-year-old female with a medical history of spinal fractures at the L5, S1, and S2 levels following a fall two years ago presented to the emergency department. She had previously undergone lumbar fusion but reported worsening symptoms, including severe chronic lumbar pain radiating to both legs and hips, saddle numbness, and worsening fecal incontinence. She denied having fever, cough, abdominal pain, dysuria, hematuria, or blood in her stool. During the physical examination, she exhibited full strength (5/5) in the upper extremities, while her lower extremities showed slightly reduced strength (4/5) due to pain in the hips, groin, and lower back. Sensation was intact for both superficial and deep pain, as well as tactile discrimination. Reflexes in the upper and lower extremities were intact (1/4). Although urinary sphincter control was preserved, the patient reported worsening fecal incontinence. Lumbar tenderness was noted. 

**Figure 4:**
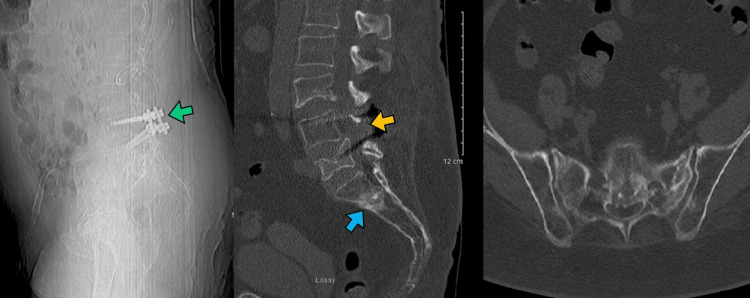
A lateral X-ray in the first image indicates a previous bilateral transpedicular fixation at the L4 and L5 levels (green arrow). The CT scan is shown in a sagittal view in the second image and in an axial view in the third image. It also reveals a prior laminectomy at L4 (orange arrow). Additionally, there are visible signs of fractures at the inferior endplate of S1 and the superior endplate of S2 (blue arrow), extending bilaterally.

**Figure 5:**
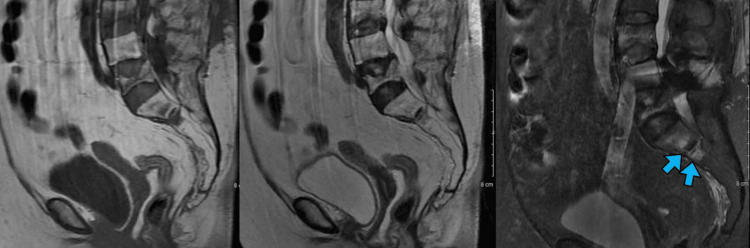
T1 (first image), T2 (second image), and STIR (third image) images in sagittal view. The STIR sequence reveals enhanced visualization of hyperintensity at the inferior endplate of S1 and the superior endplate of S2 (blue arrows), consistent with sacral insufficiency fractures. STIR, short tau inversion recovery.

An X-ray and CT scan of the lumbar spine (Figure 4) revealed postsurgical changes from the prior fusion, including bilateral pedicle screws at the L4 and L5 levels. There was a Schmorl’s node with moderate compression deformity of the interior endplate at the L3 vertebral body and a moderate-to-severe compression deformity of the L5 vertebral body, along with mild anterolisthesis of L4 on L5. Additional findings included calcifications of the abdominal aorta and a suspected parapelvic cyst on the left side. The imaging also showed a displaced linear defect in the left sacrum, medial to the sacroiliac joint, with degenerative changes in the sacroiliac joints and mildly displaced bilateral sacral fractures, suggesting recent fractures. Lumbar and sacral MRI were performed (Figure 5). 

**Figure 6:**
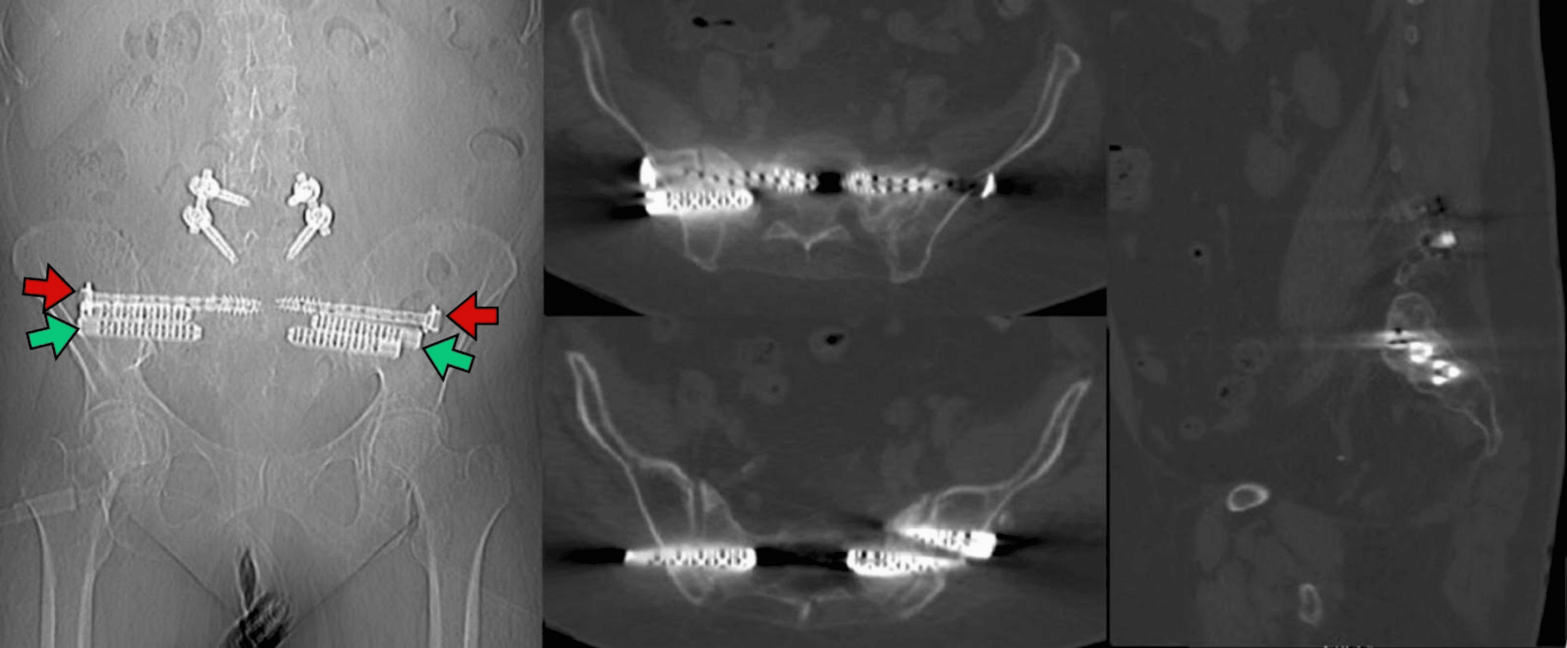
Details the surgical treatment received, including a panoramic X-ray (first image) that shows bilateral trans-sacral iliac screws (red arrows) and two screws for bilateral sacroiliac fusion (green arrows). Both axial and sagittal CT images (second and third images) confirm correct screw placement.

The patient subsequently underwent a bilateral sacroiliac joint fusion with trans-sacral screw fixation. Postsurgical imaging was done (Figure 6).

## Discussion

Sacral anatomy and its relationship with SIF

The sacrum is formed by five fused vertebrae that resemble an inverted triangle, with a sacral canal that continues from the vertebral canal and ends at the sacral hiatus. The spinal cord ends at the levels of L1-L2, but the dura mater extends down to the S2 level. The sacrum below S2 is not essential to deambulation or the support of the spinal column. Although the sacrum below the S2 level is not essential for ambulation or primary spinal support, this region may become unstable, as pressure may be exerted during sitting or in the supine position, potentially resulting in pain [17,18]. 

**Figure 7:**
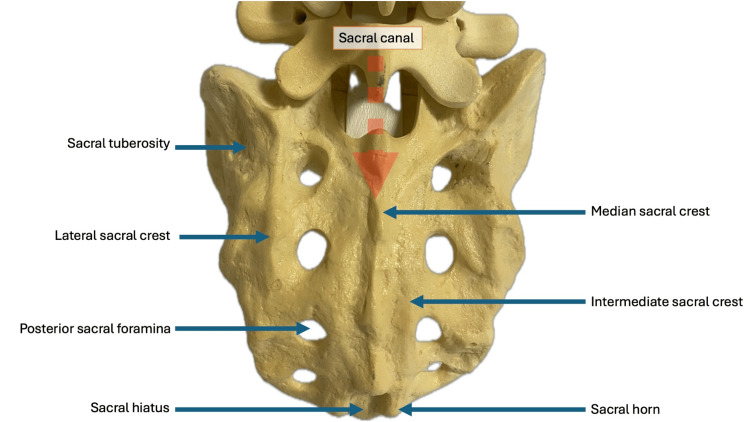
Dorsal view of the sacrum. Image credit: The authors, using an original anatomical model and edited on Microsoft PowerPoint (Microsoft Corporation, Redmond, WA, USA).

**Figure 8:**
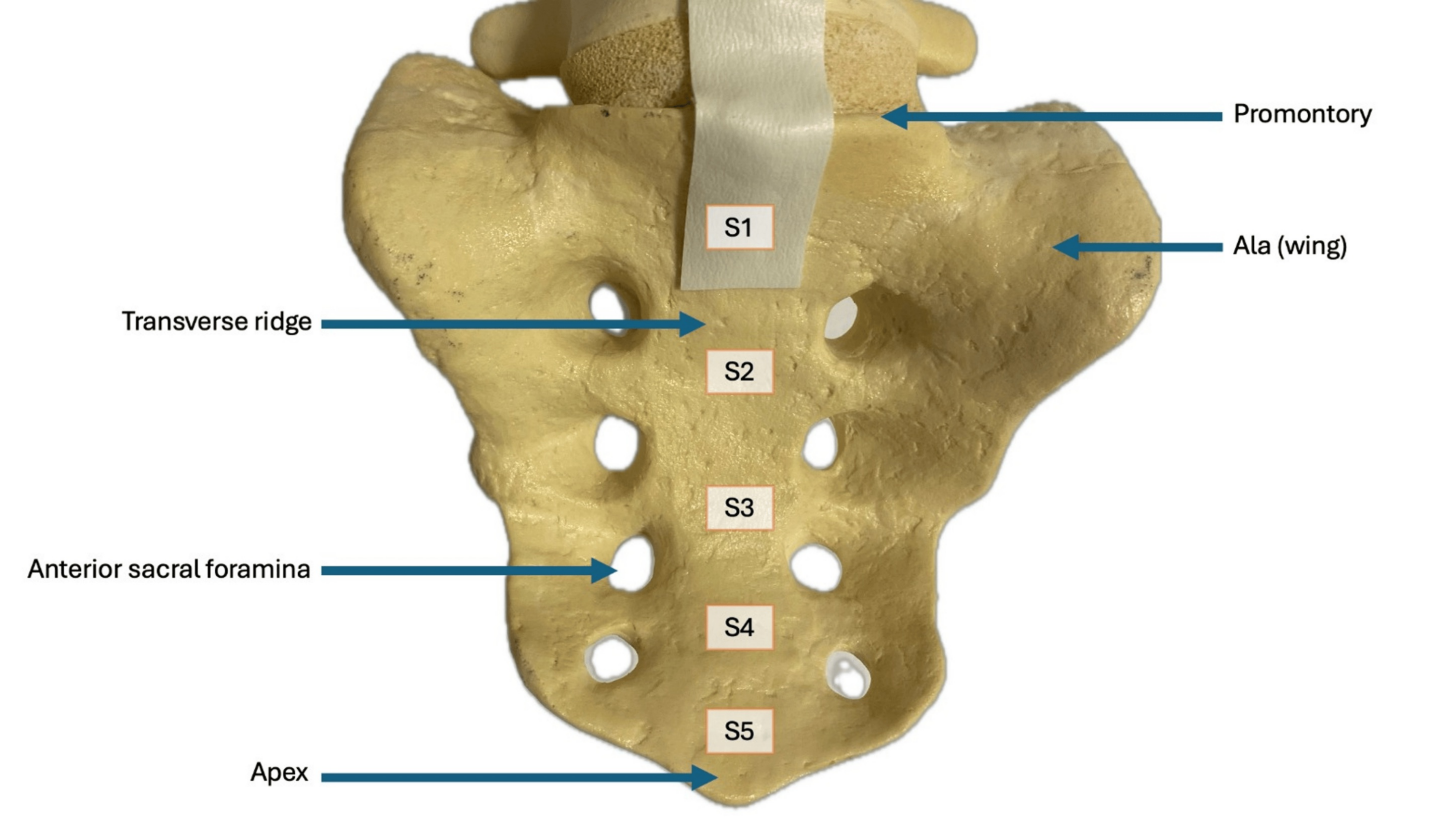
Ventral view of the sacrum. Image credit: The authors, using an original anatomical model and edited on Microsoft PowerPoint (Microsoft Corporation, Redmond, WA, USA).

The sacral canal houses the filum terminale and four pairs of sacral foramina (S1-S4) (Figure 7); fractures in these regions can cause neurological symptoms. The sacral promontory connects with the L5 vertebral body, forming the lumbosacral joint (Figure 8). The alae articulate with the ilia at the sacroiliac joints, which allows for the transfer of forces from the lower limbs to the vertebral column [18,19]. 

The posterior surface of the sacrum displays several important bony landmarks: The median sacral crest, the lateral sacral crests, and the sacrotuberous ligaments. In contrast, the anterior surface of the sacrum features four transverse ridges that are remnants of fused intervertebral discs. Overall, this structure provides a sturdy base that supports the weight of the entire body [18]. 

There are five pairs of sacral spinal nerves (S1-S5). S1-S4 nerves originate from the cauda equina and exit through the four sacral foramina; the S5 nerve instead exits through the sacral hiatus. The vertebrae receive innervation from the meningeal branches of these spinal nerves [18,20,21].

Surgical rationale

In contrast to nonsurgical treatment, surgical fixation typically leads to immediate symptom relief, improves mobility, durably reduces pain, and is associated with a lower rate of treatment failure with little risk of complications [16,22-25].

The two patients presented as examples were treated with surgical fixation from this perspective. This procedure is currently performed percutaneously, which reduces operative time and minimizes perioperative and postoperative complications. Pain relief is immediate, resulting in good short- and long-term outcomes [24]. The two patients did not improve with conservative treatment, and it is noted that this type of management may take 9-12 months for complete resolution, which could lead to systemic complications [25].

Sacroplasty has demonstrated effective pain relief and a quick return to mobility; however, there are continuing concerns of cement leakage, particularly near nerve roots. This is especially relevant for patients with open fracture lines that extend into a sacral foramen or the dural canal [22,24,25]. In our two cases, the SIFs affected both S2 regions, extending to the edges of the foramen as illustrated in the MRI results. We also believe that sacroplasty does not adequately address the primary cause of pain, which is instability. Therefore, we recommend surgical fixation as the preferred approach; it provides definitive stabilization of these fractures and resolves the associated symptoms.

Risk factors

One of the most common risk factors when it comes to SIF is osteoporosis, specifically in the older population [26]. The mortality rates for SIF remain unclear; however, a study by Park et al. [27] aimed to investigate this impact by analyzing the mortality rate of patients with SIF and determining the risk factors correlated to these mortality rates. The mortality rates were as follows: 9.8% at six months, 17.5% at one year, and 25.5% at three years. Significant factors associated with higher mortality included male gender, a history of malignancy, lumbosacral fusion extending to the S1 level, prior stroke, low total femoral bone mineral density, and a low body mass index [27,28]. Additional risk factors include rheumatoid arthritis, long-term corticosteroid therapy, postmenopausal status, and being older in women [29]. 

Based on the risk factors outlined in the literature, the primary risk factors for our patient are age and gender. While osteoporosis was not confirmed through densitometry testing, the CT scan revealed indirect signs of reduced bone density. Previous reports have noted similar cases with few identifiable risk factors [30].

Symptoms

While symptoms may be nonspecific, it is crucial to consider risk factors that can raise suspicion for this condition. Patients with SIF typically present with pain in the lower back, hips, and buttocks. Due to the nonspecific nature of these symptoms, diagnosing SIF can be difficult, especially since it can resemble other conditions, such as lumbar spinal canal stenosis [31].

Neurological involvement in SIF is present in 25% of patients, ranging from minor neuropraxia to cauda equina transection. Some researchers suggest a link between SIF and parasymphyseal discomfort [32].

Because of the nonspecific nature of SIF symptoms, such as lower back pain, sacral pain, hip or pelvic pain, pain exacerbated by movement or weight-bearing, and sciatica-like symptoms, and how similar the condition can mimic other diagnoses, documented specific symptoms are limited. There is a need for further research to better characterize symptoms of SIF and improve clinical awareness of the condition [23].

Diagnosis

SIF is very commonly misdiagnosed or missed due to the various nonspecific symptoms mentioned. Physical examinations may depict normal results, yet some patients may have decreased ankle reflexes or diminished back extension. In such cases, X-ray findings may also come back unremarkable. Sacral radiographs are delayed to 40-55 days, and only 20-38% of SIFs are depicted on X-ray, with only 12.5% showing visible fracture lines [4,6,9,11]. It is important to get an early diagnosis, as a delayed diagnosis can lead to further immobilization and cause further complications like thrombosis and cardiac issues. Because of its golden standard in diagnosing acute traumatic fractures, many providers have resorted to using CT scans. Nonetheless, SIF has no such standard identified, and CT scans can even fail in fracture detection if there is overlying cortical bone or microfractures that cannot be well-visualized. Therefore, MRI has been pronounced the superior diagnostic technique for occult SIF [33].

In a study by Kinoshita et al. (2019), MRIs were able to correctly diagnose 74% of patients with known SIFs. Bone scintigraphy, another technique that is used to diagnose SIF by depicting an "H" sign characteristic in patients who have the condition, was also used in one patient. Bone scintigraphy was not recommended over MRIs, as it is more invasive since it utilizes radiation exposure [34].

Yamauchi et al. (2022) found that MRIs with fat suppression images had the most sensitivity (100%), compared to CT (94.5%) and X-rays (24.2%). MRIs were also able to identify not only SIF, but also associated fractures/pathologies, such as pelvic fractures and spine fractures [12]. 

However, despite having a higher sensitivity, CT scans are quicker, have better visualization of fracture lines, are required for surgery planning, are less expensive, and are more easily available. Therefore, it is important to note that if an SIF is diagnosed with an MRI and surgery is planned, further evaluation with a CT of the pelvis is still recommended [33].

Our cases demonstrate the importance of MRI in diagnosing SIF, as it provided a clearer visualization of the fracture that was not well seen on X-rays and CT scans.

Denis classification

**Figure 9:**
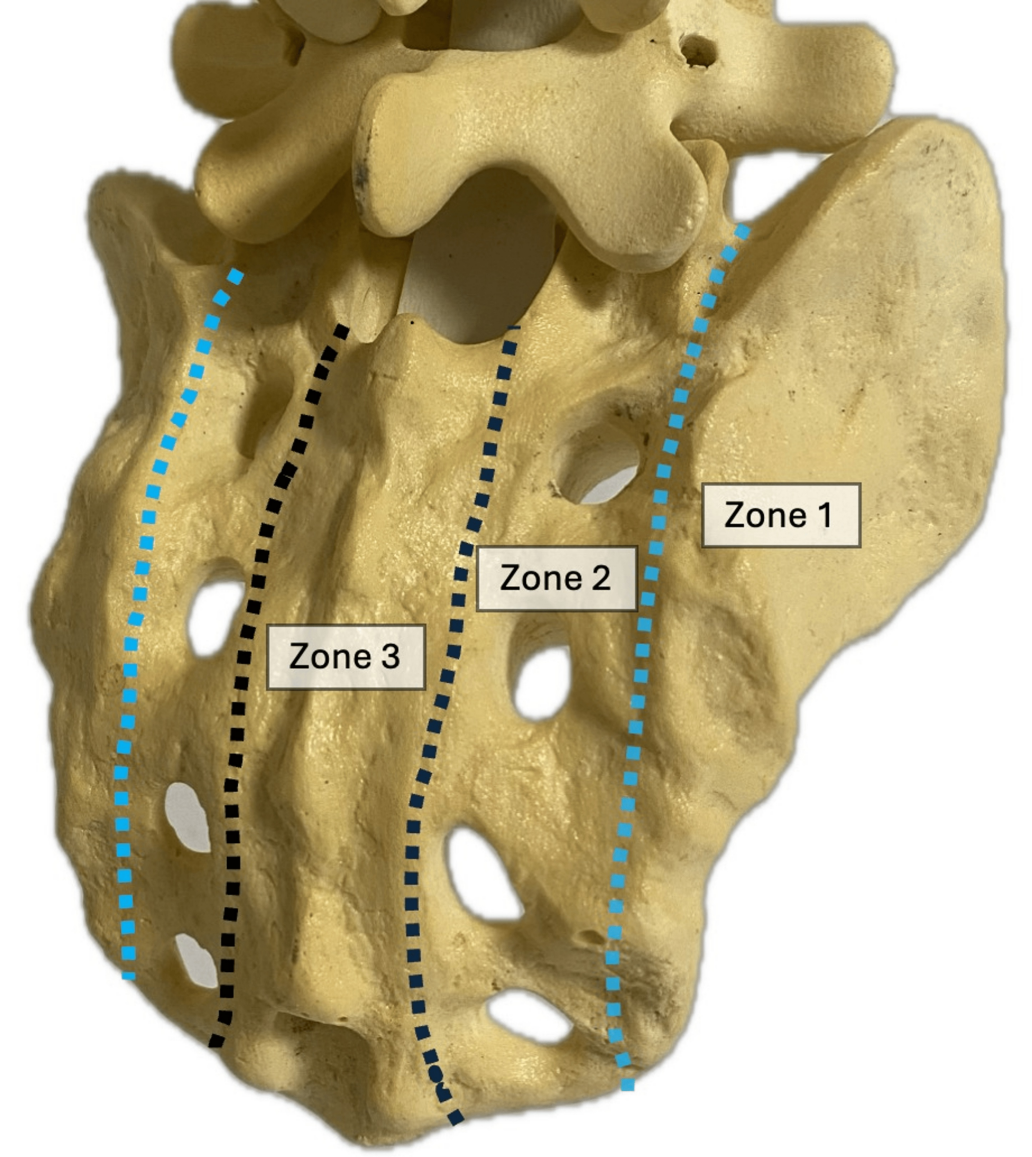
Denis classification. Image credit: The authors, using an original anatomical model and edited on Microsoft PowerPoint (Microsoft Corporation, Redmond, WA, USA).

Denis et al. categorized the sacrum into three anatomical zones (Figure 9): Zone 1, the sacral wing region occasionally associated with L5 root injury and is the most prevalent, accounting for approximately 50% of such cases, and the reduction of the ala promotes L5 recovery. Zone 2, the sacral foramen region associated with unilateral L5, S1, and S2 nerve injury and rarely with bladder dysfunction, accounts for 34% to 47.5% of all sacral fractures, and reduction and fixation may improve neurologic deficits. Zone III affects the sacral canal region, frequently associated with sphincter dysfunction and saddle anesthesia, and is subdivided into vertical and horizontal fractures; reduction and decompression are controversial as they don’t ensure recovery. U-shaped sacral fractures are unstable and have to be stabilized with percutaneous fixation [35,36].

Treatment

The first line of treatment for SIF is conservative [21]. These include bed rest, rehabilitation, analgesics, and mobilization using walkers or crutches. Conservative management is usually recommended for up to six months (it can range from eight weeks to two years for complete resolution). However, prolonged immobilization can lead to complications like deep vein thrombosis, which can create challenges in conservative treatment. Additionally, some patients may experience persistent pain that limits their daily activities [37,38]. Intraarticular corticosteroids can reduce pain rapidly, but they are also associated with tissue damage and systemic complications and have a reported failure rate of 26%. Biological alternatives like autologous platelet-rich plasma have shown pain improvement with regenerative properties, especially after six months of treatment, though it has a reported failure of 14%. Hyaluronic acid improves synovial viscosity and joint lubrication, used particularly in degenerative conditions [39-42]. SIF is particularly seen in elderly women with osteoporosis; in those cases, osteoanabolic agents such as teriparatide, romosozumab, and denosumab have been in use to increase bone formation, and their side effects include headache, hypercalcemia, hypercalciuria, and dizziness; further studies are still needed to assess the efficacy in the setting of fracture healing [22,43,44].

**Figure 10:**
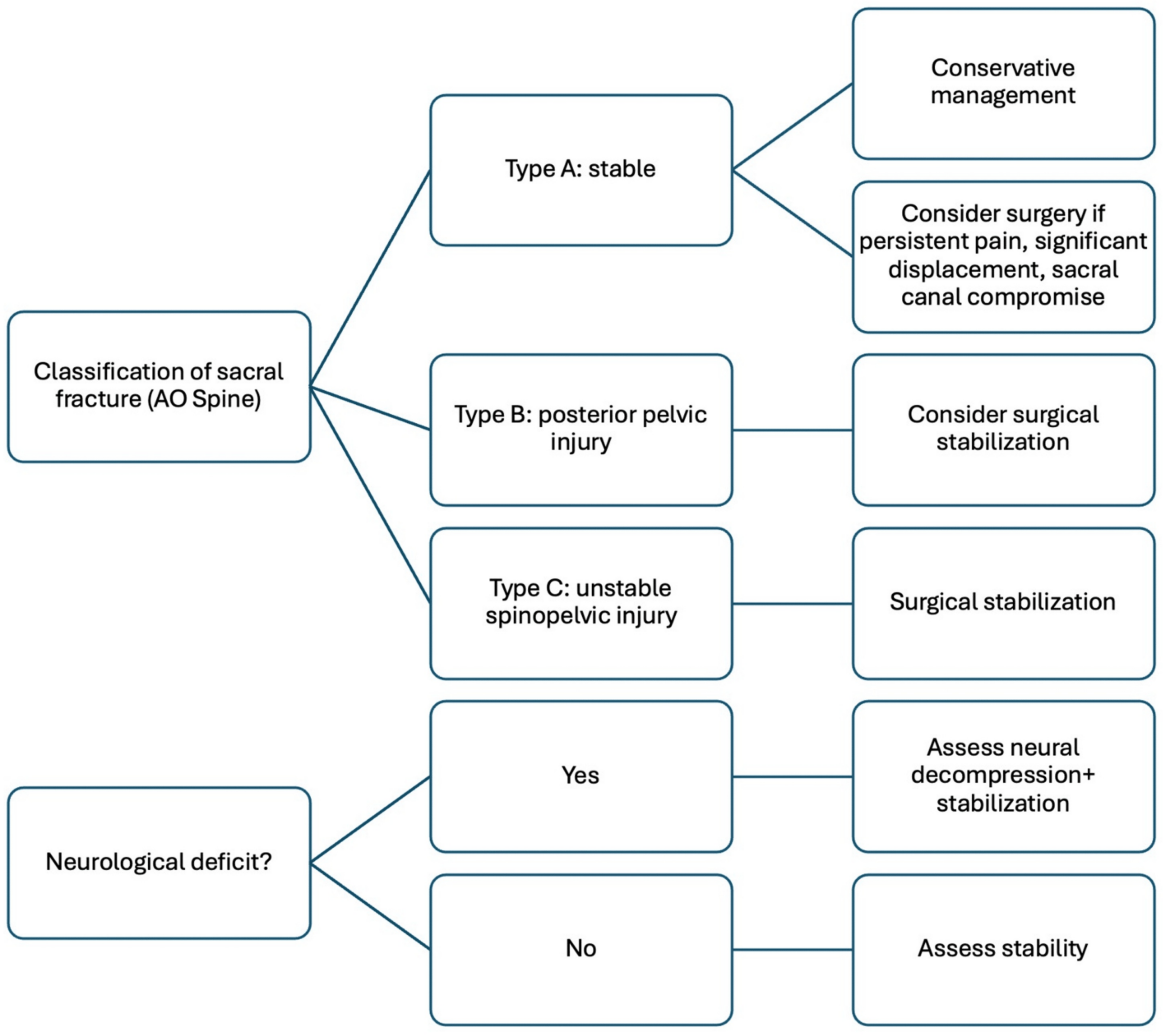
Management algorithm for sacral insufficiency fractures incorporating neurological status and Denis classification. Image credit: The authors, using Microsoft PowerPoint (Microsoft Corporation, Redmond, WA, USA).

Nevertheless, when conservative treatments fail, operative approaches are recommended (Figure 10) [22]. Surgical treatment is successful in pain control and may improve radicular or sphincter deficits, as demonstrated in a systematic review where 65% of patients received sacropelvic fixation, with 94% of cases achieving symptom resolution or overall pain reduction [23]. 

Denis classification (Figure 10) is focused on sacral fracture morphology; biomechanical stability and the neurological status of patients can also be studied under the AO spine sacral and pelvic classification. 

Type A consists of lower sacrococcygeal injuries, and they do not produce instability of the posterior pelvic or spine. The A1 subtype includes coccygeal compression or ligament avulsion. The A2 subtype is nondisplaced transverse fractures below the sacroiliac joint. The A3 subtype is displaced transverse fractures below the sacroiliac joint. The type B fractures are in the posterior pelvis area, and usually they need surgical repair. The B1 subtype corresponds to the previously type III Denis injuries involving the spinal canal. The B2 subtype is transalar fractures that do not involve foramina or the spinal canal. The B3 subtype involves the foramina but not the spinal canal. Finally, type C fractures are spinopelvic injuries and are the most unstable. The C0 subtype is a nondisplaced U-fracture, and the C1 subtype is also a U-type fracture with a fracture line medial to the S1 facet. C2 subtype is a bilateral B injury without transverse fracture, and the C3 subtype is a displaced U-type sacral fracture resulting in dissociation of the sacrum and pelvis, generally leading to severe neurological deficits. Stabilization of unstable fractures may minimize the risk of further displacement and prevent exacerbation of neurological deficits. This system takes into consideration the neurological status and modifiers [45-47].

Age is an important factor in the decision for surgical management. In 46 patients with SIF, surgical treatment was recommended due to the severity of the fracture, yet, because they were geriatric patients, conservative treatment was implemented instead. Conservative management led to enhanced mobility without complications, despite the subjects not receiving surgical treatment. Overall, more research is needed to compare surgical and conservative approaches to assist providers in determining which treatment is most effective in various patient groups. Treatment decisions should be individualized and interdisciplinary, based on patient demographics, location, and severity of fracture [41]. In our case, we decided to pursue surgical treatment, despite the patient’s age, because the severity of the symptoms was hindering rehabilitation. We opted for a minimally invasive technique, performing bilateral sacroiliac fusion with a trans-sacral screw. This approach successfully stabilized the sacral fracture.

## Conclusions

SIFs are often not diagnosed early enough because they have nonspecific symptoms and subtle radiographic findings. They mainly affect elderly patients with osteoporosis. MRI is the best way to diagnose SIFs as it is the most sensitive diagnostic tool, and early detection is crucial to prevent complications. Conservative management is the first treatment option, but if it fails, surgical intervention like a trans-sacral screw can provide significant pain relief and improved function. The surgical option should not be dismissed in elderly patients, as minimally invasive techniques are now available to effectively stabilize complex fractures with a lower risk of morbidity and mortality.
